# Behaviour and Locomotor Activity of a Migratory Catostomid during Fishway Passage

**DOI:** 10.1371/journal.pone.0123051

**Published:** 2015-04-08

**Authors:** Ana T. Silva, Charles Hatry, Jason D. Thiem, Lee F. G. Gutowsky, Daniel Hatin, David Z. Zhu, Jeffery W. Dawson, Christos Katopodis, Steven J. Cooke

**Affiliations:** 1 Fish Ecology and Conservation Physiology Laboratory, Department of Biology, Carleton University, Ottawa, Ontario, Canada; 2 Ministère des Forêts, de la Faune et des Parcs, Longueuil, Québec, Canada; 3 Department of Civil and Environmental Engineering, University of Alberta, Edmonton, Canada; 4 Department of Biology, Carleton University, Ottawa, Ontario, Canada; 5 Katopodis Ecohydraulics Ltd., Winnipeg, Manitoba, Canada; University of Girona, SPAIN

## Abstract

Fishways have been developed to restore longitudinal connectivity in rivers. Despite their potential for aiding fish passage, fishways may represent a source of significant energetic expenditure for fish as they are highly turbulent environments. Nonetheless, our understanding of the physiological mechanisms underpinning fishway passage of fish is still limited. We examined swimming behaviour and activity of silver redhorse (*Moxostoma anisurum*) during its upriver spawning migration in a vertical slot fishway. We used an accelerometer-derived instantaneous activity metric (overall dynamic body acceleration) to estimate location-specific swimming activity. Silver redhorse demonstrated progressive increases in activity during upstream fishway passage. Moreover, location-specific passage duration decreased with an increasing number of passage attempts. Turning basins and the most upstream basin were found to delay fish passage. No relationship was found between basin-specific passage duration and activity and the respective values from previous basins. The results demonstrate that successful fishway passage requires periods of high activity. The resultant energetic expenditure may affect fitness, foraging behaviour and increase susceptibility to predation, compromising population sustainability. This study highlights the need to understand the physiological mechanisms underpinning fishway passage to improve future designs and interpretation of biological evaluations.

## Introduction

Worldwide, fishways are an integral and growing component of projects designed to restore river longitudinal connectivity and to facilitate upstream passage of migrating fish. Although they represent a potential solution to reestablish fish migratory routes, successful ascension by fish may require levels of swimming activity which result in high energetic expenditures [1]. Though efforts have been developed to link species-specific swimming ability with fishway design [2,3], few efforts have been made to understand the physiological costs and consequences of fish passage [4,5], even though the use of behavioural and physiological knowledge to inform resource management and conservation strategies [4] has great potential for applied fisheries issues.

Locomotion in aquatic systems is challenging as result of high friction and resistance imposed on a body moving through water and the associated energy lost in the wake during propulsion [6]. Swimming activity can account for a considerable portion of the energy budget of a fish [7], up to 40% of the total [8]. Thus, migratory movements can be energetically demanding [9] and may result in a significant energy loss, particularly in species that cease feeding. During migration fish face challenging hydraulic conditions that often require fish to make behavioural adjustments that affect energetic expenditure [10]. Depending on the time or energy available fish may then adopt different energetic optimization strategies. If energy is a limitation, the energy optimization strategy (i.e. fish swim slowly and take more time searching for lower velocity areas in order to save energy) may be preferable; a time optimization strategy (i.e. fish swim quickly through higher velocity areas) may be more suitable when energy is not a constraint [11].

In the past decade, the biologging community has developed a variety of highly sophisticated techniques enabling researchers to study the behaviour and physiology of free-swimming fish [12]. These biologging tools are particularly important as they can provide insights into the relative swimming effort and energetic expenditure associated with different animal activities and their movement through particular landscapes [13]. The allocation of time and energy to different behaviours affects individual survival and fitness [14,15] strongly determining the life-history strategies of animals. Although measuring the energetic status of animals is a key component to understanding how they interact with their surrounding environment [16] and humans [17], there are many challenges with estimating energy expenditure from free-living animals in the wild [18]. Recently, accelerometry, which relies on the relationship between body acceleration and energy expenditure [19,20], has emerged as an effective biologging option for estimating the rate of energy expenditure under field conditions. Animal movement is generally achieved through muscle contraction, which leads to body and/or limb acceleration, which is correlated with energy expenditure [19,21]. Acceleration measured using triaxal accelerometers (electromechanical device composed of three orthogonally mounted uniaxial piezoresistive accelerometers that can be used to register accelerations, [22]) represents the summation of two components; acceleration due to gravity (static acceleration) and acceleration from the motion of the animal-borne device (dynamic acceleration) [23,24]. Overall dynamic body acceleration (ODBA) results from removing static acceleration from the acceleration logger data, and is described as the total dynamic acceleration in the center of mass of the animal as a result of the movement of body parts in all three dimensional axes [25,26]. The use of ODBA relies on the principle that energy use increases with active movement and has been shown to correlate linearly with energy expenditure as measured by the rate of oxygen consumption in different invertebrates (e.g. lobster and scallop) [25,27] and vertebrates species (e.g. imperial shag, penguins and turtles) [28], therefore, ODBA is deemed a good proxy for the determination of field metabolic rate in animals [25,29,30,31, 32, 33]. Physiological tools are being used increasingly in hydropower assessments [34] and more generally to provide information on organism health and condition to inform conservation and management initiatives (i.e. conservation physiology) [35], yet there is a lack of information on fish behaviour and physiological processes which underpin interactions between fish and hydraulics in fishways that guide fine-scale swim path selection that potentially influences fish passage duration and success [36,37]. Fish use different physiological mechanisms depending on whether time minimization or energy maximization is more pertinent [38]. Although the use of activity metrics such as ODBA cannot address how the physiological mechanisms evolve in response to the constraints on both time and energy, ODBA measures the variation in locomotion-related power requirements at a fine temporal scale, providing insight into the allocation of effort in different ecological situations and thereby the energy used over a range of spatial and temporal scales.

Silver redhorse (*Moxostoma anisurum*) is an iteroparous catostomid with a relatively limited capacity for swimming and poor metabolic recovery response when compared with other species such as salmonids or even other redhorse species (*Moxostoma* spp.) [39]. Catostomid species are obligate migratory fish which frequently dominate fish abundance and biomass in North American rivers [39]. Migratory routes of catostomids are often fragmented by artificial barriers, which may negatively affect their populations [40]. Although fishways may facilitate free migration of catostomids, these structures may also impose a particularly high energetic expenditure for these species. Thus, information about performance of fish species in dynamic hydraulic environments (such as fishways) can elucidate the way in which animals react to and are impacted by various hydraulic conditions. Nonetheless, physiological data, which can be used to determine whether the impediments to passage success are behavioural or related to physiological capacity, are often lacking [41].

This study analyzes the behaviour and physiology of migrating silver redhorse during fishway passage by combining location-derived information from a passive integrated transponder (PIT) array, with biologging accelerometers to investigate activity of fish during upstream passage. Specifically, we were interested in: 1) quantifying the swimming effort of silver redhorse during upstream fishway passage, 2) determining whether time spent in each basin, groundspeed (GS) and activity differ among locations within the fishway, 3) identifying biological, behavioural and structural variables that influence fish activity during fishway passage.

## Materials and Methods

### Ethics Statement

Scientific Collection Permits were provided by the Ministère des Forêts, de la Faune et des Parcs du Québec. None of the research efforts involved imperiled species. All animal trials and sampling were conducted in agreement with national and international guidelines to maintain the welfare of experimental animals. Surgical and handling protocols were reviewed and approved by the Canadian Council on Animal Care administered by the Carleton University Animal Care Committee (B10-12). All efforts were made to minimize stress during capture and tagging. No fish were sacrificed to complete this study.

### Study site

**Fig 1:**
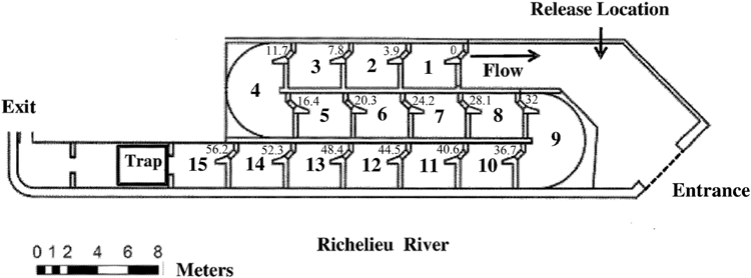
Schematic of the Vianney-Legendre vertical slot fishway on the Richelieu River in Quebec, Canada. Distance metrics, corresponding to locations of PIT antennas, indicate the cumulative minimum transit distance between successive fishway basins (beginning at 0 m). The order of the basin starts from downstream (basin 1) to upstream (basin 15) in accordance with the direction of fish movement.

This study was undertaken at the Vianney-Legendre Fishway, a vertical slot fishway located on the Richelieu River adjacent to the St. Ours dam (45°03051'48"N, 73°03008'60"W) in southwestern Quebec, Canada. The fishway is an 85 m long concrete structure with an elevation rise of 2.65 m and an average slope of 4%. The fishway is divided into 13 regular rectangular basins (3.5 m long × 3.00 m wide) connected by two resting/turning basins with curved walls (2.75 m radius) (Fig 1). Each pool is equipped with a 0.60 m wide vertical slot (b_0_) (2.30 to 4.00 m height range) and the head drop between consecutive pools (Δh) is 0.15 m. This corresponds to a potential velocity (V_s_) of 1.72 m.s^-1^ based on the calculation from the formula V=s2gΔh = 1.72 m.s^-1^, where *g* is the acceleration due to gravity (9.81 m.s^-2^). The flow in the fishway is non-uniform among basins, with different velocity levels and velocity patterns as a result of the small difference in the ratio between the slot size and basin size (5.75b_0_ length × 4.93b_0_ width) of the regular basins. The flow discharge capacity of the fishway is 1 m^3^.s^-1^ with additional potential for 6.50 m^3^ s^-1^ attraction flow released at the entrance that was not used during this experiment. More detailed information on the fishway is provided in Thiem et al. 2011 [42].

### Fish collection and tagging

Nineteen silver redhorse (mean total length (L_t_) ± S.D. = 555 ± 33 mm, mean total weight (W_t_) ± S.D. = 2.18 ± 0.51 kg) were captured between 20 April and 5 May, 2012 by means of a rectangular trap (2.2 × 2.0 m cage) with an entrance width of 0.28 m installed at the upstream end of the fishway. The trap was built of galvanized steel with a shade mesh floor to prevent damage to fish during the raising process, had a horizontal bar spacing of 38 mm and was raised using an electric winch. It was not possible to capture fish downstream using nets or electrofishing given the presence of federally endangered copper redhorse (*M*. *hubbsi*) and concerns regarding their incidental capture. We assumed that capture in the fish trap of large numbers of silver redhorse indicated optimal timing of migration and thus individuals were tagged as encountered. A passive integrated transponder (PIT) array consisting of 15 complete pass-through antennas (beginning at antenna 1 downstream (0 m) and ending at antenna 15 upstream (56.2 m) was installed within the fishway (Fig 1). The antennas were scanned sequentially at high speed (2.5 times per second) and upon positive detection a unique tag identification number, antenna number and date and time stamp were stored by a data logger (Oregon RFID, Oregon, USA).

Silver redhorse were tagged with triaxial accelerometers (model X6-2, 25 Hz recording frequency or model X6-2mini, 20 Hz recording frequency; Gulf Coast Data Concepts, Waveland, MS) at the base of the dorsal fin and with PIT tags (23 x 3.85 mm HDX: Texas Instruments, Dallas, USA) injected into the coelomic cavity. Radio tags (149 MHz, 8 g weight in air, burst rate 2 s, 90 day battery life, Sigma Eight Inc., Newmarket, Ontario, Canada) were also attached to accelerometers to facilitate tag retrieval at the end of each trial. External accelerometer attachment was accomplished using 6 gauge hypodermic needles, and 20 gauge stainless steel wire to secure tags. Tagging methods were similar to those previously used on several species [43]. The same experienced tagger attached all tags. Fish were tagged in V-shaped troughs with flow through ambient river water supplied during each tagging event. The combined mass of the tags was 38 g (representing 1.3–3.0% of the total body weight of fish), in some cases exceeding the general 2% tag burden rule [44], although well below the 12% tag burden previously demonstrated to affect swimming performance in juvenile rainbow trout (*Oncorhynchus mykiss*) [45]. Holding tanks for recently tagged fish had a constant supply of fresh river water. In all cases, tagging occurred with the head and gill complex of fish submerged in fresh water to minimize stress and fish required only light restraint administered by the individual holding the fish during tag attachment. Anesthetics were not used given the assumption that there would be a protracted behavioural recovery period and based on earlier experience where it was determined that some redhorse species did not respond well to anesthesia [46]. Once tagged, fish were released into the entrance basin of the fishway (Fig 1) in three separate groups to minimise the number of redhorse in the fishway at any time whilst maintaining adequate sample sizes. Trials ran for 72 h whereby individual fish were able to volitionally ascend the fishway (Trial 1 (n = 7): 20–23 April, mean water temperature 9.2 ± 0.1°C; Trial 2 (n = 5): 28 April-1 May, mean water temperature 7.8 ± 0.0°C; Trial 3 (n = 7): 4–7 May 2012, mean water temperature 10.4 ± 0.1°C. A block net prevented downstream passage out of the fishway and installation of the fishway trap prevented upstream escape. Fish were recaptured following a slow dewatering of the fishway at the end of each trial, accelerometers removed and fish released.

### Data analysis

From the 19 tagged fish, accelerometer data information of nine fish was not usable due to tag electronic components being water damaged. To standardize procedures, analyses were limited to fish that successfully ascended the fishway (n = 9) and the remaining individual was excluded from further analysis. Fish movements through the fishway were reconstructed by converting PIT antenna locations to distance metrics beginning at the first antenna and ending at the most upstream vertical slot. A passage attempt was defined as any movement into the fishway (a PIT record to at least the second antenna encountered) and terminated upon either successful passage or return to the downstream staging area. Fish detected on the most downstream antenna were considered to be inside the fishway and successful passage was defined as the first detection of an individual on the most upstream antenna (immediately downstream from the fish trap). Passage duration and time in each basin were determined from PIT data for each individual fish. Total fishway passage time was calculated as the time taken from the last detection on the most downstream antenna to the first detection on the most upstream antenna, whereas time in each basin was calculated as the time taken from a fish’s last detection on the most downstream antenna of the basin to the first detection on the most upstream antenna of the respective basin. Accelerometer output for each tag was time calibrated with the PIT antenna system manually by applying a linear correction factor to account for time drift between systems. A single accelerometer output in *g* (gravitational units) was divided into relevant static and dynamic acceleration components using a weighted smoothing interval of 1.5 s in Igor Pro (version 6.0, WaveMetrics Inc., Lake Oswego, Oregon, USA). Static acceleration was subtracted from total acceleration in each individual axis to yield dynamic acceleration. Absolute values of dynamic acceleration from each acceleration axis were summed to yield instantaneous Overall Dynamic Body Acceleration (ODBA) [31].

### Statistical analysis

To test the hypothesis that the time of passage of the fishway was equivalent among fish, a Kruskal—Wallis ANOVA was employed. To determine location-specific differences in 1) time in basin (TIB), 2) groundspeed (GS, herein defined as the ratio between the total length of the basin and the time to pass a basin) and 3) ODBA among fishway locations (basins), data were first plotted against predictors including basin number, fishway passage attempt, fish ID, body size (L_t_), time spent in previous basin (TIPB), groundspeed in previous basin (GSPB), and median ODBA in previous basin (ODBAPB).

Median time in basin was fitted with a Generalized Linear Mixed Model (GLMM) that included fish ID as a random factor. Preliminary GLMMs on the response variables GS and ODBA indicated strong non-linear patterns in the residuals vs the fitted values, thus these data were fitted with Generalized Additive Mixed Models (GAMMs) using basin number (1–14) as a smoothing function [47] and fish ID as a random factor. Data were analysed using the nlme function implemented in the *R* statistical environment (package version 3.1–117, R core development team 2008 [48]). To compare model fits objectively, and determine which was the most appropriate, an information theoretic approach was performed using Akaike’s information criterion (AIC; Akaike 1974) [49]. The best model was the one that possessed the lowest AIC value. Models were validated by examining histograms of the normalized residuals, plotting the normalized residuals against fitted values, response variables and predictor variables including those not in the model, and by examining residual lag-plots to assess serial autocorrelation. Given patterns of heterogeneity in the residuals, ODBA and TIB were log transformed. In addition, all models were also fitted with a variance structure to correct for heterogeneity in the residuals [47]. Again, models were validated using the techniques described above. The final models were refitted using restricted maximum likelihood [47]. Despite the strategies to generate unbiased parameter estimates, a model for GS could not be validated due to strong residual patterns. Heteroscedasticity (i.e. error terms in a model that do not have a constant variance) does not usually cause any bias in the estimated model coefficients themselves [50], but it influences the standard errors around these coefficient estimates, making any statistical inferences and predictions based on the model unreliable [51]. Thus, although GS could not be modeled, GS data are presented descriptively.

## Results

**Fig 2:**
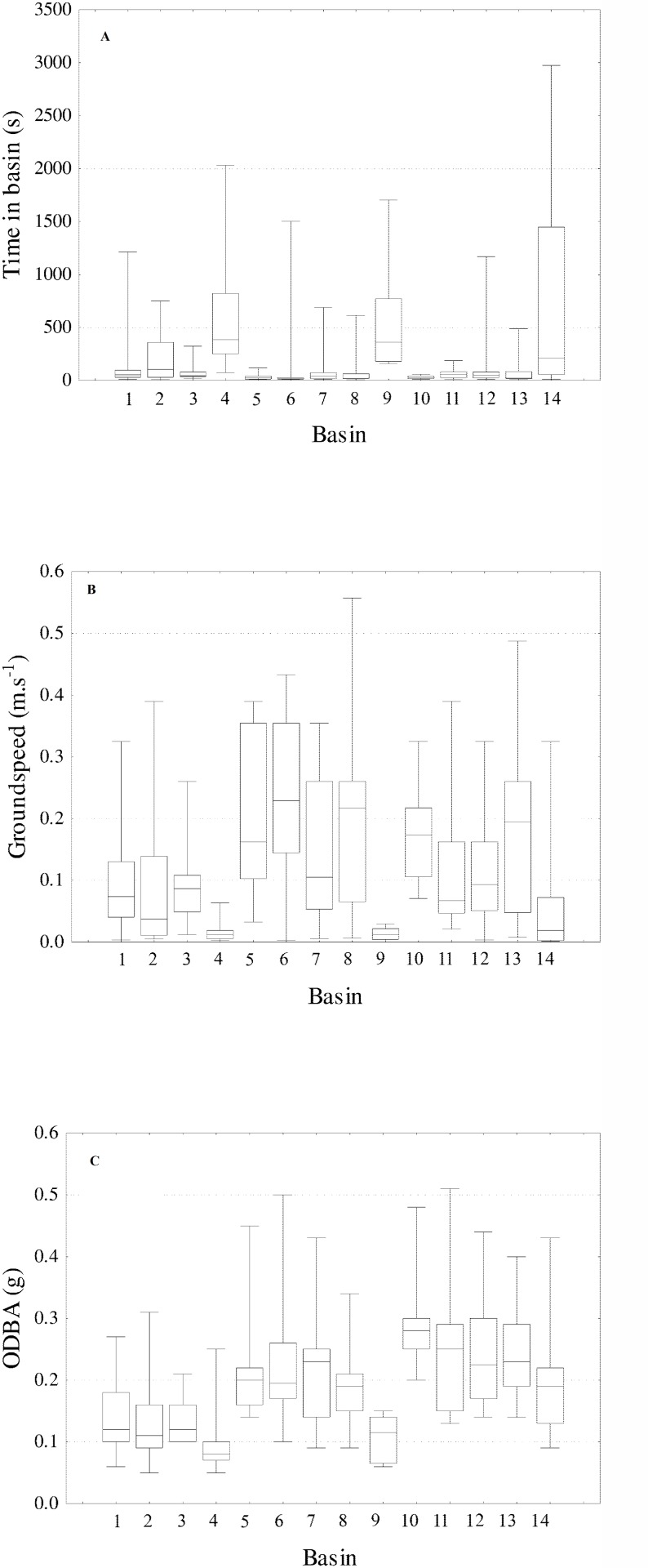
Box plots with the median (horizontal lines), interquartile ranges (boxes), and ranges (whiskers) of the time spent in each basin (A), groundspeed (B) and ODBA (C) exhibited by silver redhorse

**Fig 3:**
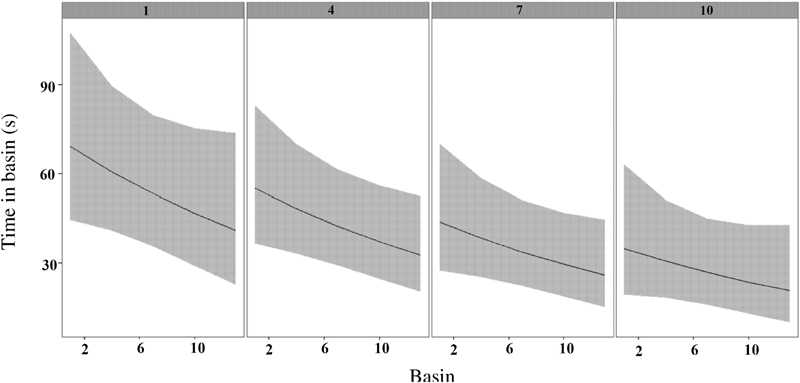
The predicted time spent in each fishway basin (seconds ± 95% confidence limits) as fish attempted pass through the fishway for the first (1), fourth (4), seventh (7), and tenth (10) time.

Silver redhorse made multiple attempts to pass the fishway (up to 12 attempts). The time taken by silver redhorse to successfully ascend the fishway was significantly different among fish (Kruskal-Wallis: *P* <0.05). TIB was also noted to vary among basins (Fig 2A), with the longest times observed in the turning basins (basin 4, median: 384 s, range: 74–2032 s and basin 9, median: 360 s, range: 159–1704 s) (Fig 2, Table 1). Furthermore fish also spent significantly longer times (median: 208 s) in the last basin (basin 14). The final model for TIB contained basin and attempt as covariates and Fish ID as a random factor (Table 1). The final model shows that individuals spent significantly less time per basin on each successive attempt to pass the fishway (Table 1, Fig 3). TIB was independent on TIPB, ODBAPB and GSPB of fish.

**Table 1:** Fixed effects from the top GLMM to explain TIB. Random intercept variance was 0.257.

Estimation method	Response	Model term	Coefficient	SE	df	t	P-value
(1)GLMM	Log(TIB)	Intercept	4.44	0.24	198	18.15	<0.001
		Basin #	-0.04	0.02	198	2.16	0.032
		N Attempt	-0.08	0.03	198	2.84	0.005
(2) GAMM	Log(ODBA)	Intercept	-1.84	0.04	203	-40.4	<0.0001
		s(Basin #)			7.143	11.7	<0.0001

Marginal (fixed-factors) and conditional (both fixed and random factors) R2 were 0.041 and 0.4, respectively. (2) The intercept and smoothing function significance in the GAMM to estimate ODBA. Random intercept variance was 0.010 and the adjusted R2 = 0.281.

During upstream passage, GS of silver redhorse were higher through regular basins in comparison to turning basins (basin 4, median: 0.01 m.s^-1^, range: 0.01–0.06 m.s^-1^ and basin 9, median: 0.01 m.s^-1^, range: 0.01–0.03 m.s^-1^) (Fig 2B). Fish were faster in the basins immediately upstream of the turning basins (basin 5, median: 0.16 m.s^-1^, range: 0.03–0.39 m.s^-1^ and basin 10, median: 0.17 m.s^-1^, range: 0.07–0.33). In general, GS of silver redhorse was highest between the first and second turning basin. After the second turning pool, fish exhibited low GS in the last basin (basin 14, median: 0.02 m.s^-1^, range: 0.01–0.33 m.s^-1^) (Fig 2B).

**Fig 4:**
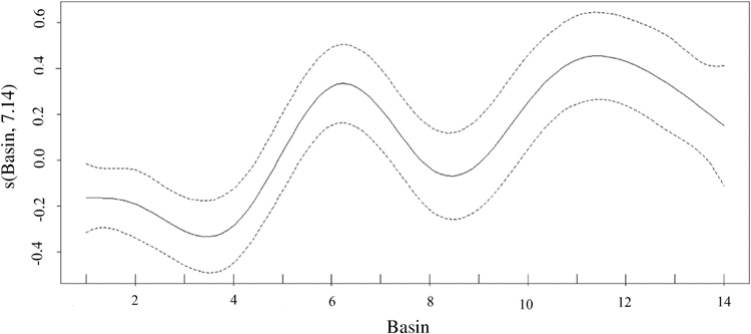
The estimated smoothing curve for fishway basin beginning at the entrance way at basin 1. The y-axis shows the contribution of the smoother to the fitted values of ODBA. The solid line is the fitted curve and the dashed lines are the approximate 95% pointwise confidence limits. Model degrees of freedom are given parenthetically in the y-axis label

During fishway passage, silver redhorse ODBA varied substantially among individuals and locations (Minimum median value: 0.05g; Maximum median value: 0.51g). Median ODBA was lower through turning basins (basin 4, median: 0.08 g, range: 0.14–0.45 g and basin 9, median: 0.11 g, range: 0.20–0.48 g) in comparison with regular basins. In addition, silver redhorse exhibited highest median ODBA in the basin immediately upstream of each turning basin (Fig 2C). Median ODBA was relatively low until the first turning basin, after which the ODBA of silver redhorse continuously increased (Fig 2C). This trend was described by the top model that included basin as a smoothing function (Table 1, Fig 4). Similar to TIB, ODBA within each basin was not related with TIPB, ODBAPB and GSPB of fish.

## Discussion

This study confirms that the hydraulic conditions of vertical-slot fishways are suitable for upstream passage of silver redhorse as only one of the ten tagged fish that entered the structure did not successfully ascend the fishway. This corroborates the results found by Hatry et al. 2014 [39], who compared the physiology and relative swimming performance of three redhorse (*Moxostoma* spp.) species with their passage success in the same vertical slot fishway. The authors identified that despite its weaker swimming capability relative to the other two species, silver redhorse had the highest passage efficiency of the three.

The present study demonstrated that the upstream passage of silver redhorse through a vertical slot fishway is associated with increasing activity (ODBA) as fish moved progressively further upstream. Given that ODBA has previously been demonstrated to be a good proxy for energy expenditure in other teleosts [52], increased passage duration may have implications for individual reproductive success and survival due to lower energy stores. Even if energy expenditure is not a limitation for successful passage in fish, or only represents a small portion of their total energetic budget, the energy expended during fishway passage may have unintended consequences on the survival of the species. For example, energy is needed to reach spawning grounds, develop gonads, and engage in spawning activity. Indeed Roscoe et al. (2011) [5], in their study on fishway passage and post-passage mortality of up-river migrating sockeye salmon found that fishway passage had a significant impact on successful spawning migration of sockeye salmon as well as it contributed to an intra-specific selectivity on the survival rate, with significantly lower values for females (40%) than for males (71%). Moreover Burnett et al. (2014) [53] also found a female-biased migration post-passage mortality associated with higher anaerobic effort of females during upstream passage compared to males.

Silver redhorse exhibited the lowest activity (ODBA) in the turning basins compared to the regular basins during fishway ascension. This may be the result of the interaction of fish with the hydraulic conditions within the turning basins, which are characterised by low water velocities, low turbulence and recirculation areas with long and wide vortices [54]. Although there are low levels of turbulence in the turning basins [54], the distribution of eddy sizes could have strongly affected fish swimming behaviour. These typical vortical structures that play a significant role in fluid flow phenomena such as momentum, mass and heat transfer [55] are known to affect fish variably depending on the eddy intensity, periodicity, orientation and scale [56] as well as fish morphology [36]. Fish swimming behaviour and swimming capacity are known to be compromised by eddies of similar size to the total length of the fish [36]. Eddies larger than the total length of a fish may induce fish disorientation and fish displacement whereas eddies smaller than fish total length may not impact fish swimming capacity as fish are able to swim steadily through the vortices [57]. This is a complex phenomenon that results from the capacity of the fish to integrate biomechanics, physiological and sensory processes. This ability allows fish to explore turbulent areas and greatly enhance propulsive efficiency by extracting energy from eddies, thus decreasing the energy expenditure required to generate thrust [57]. Indeed, this is similar to other migratory animals such as soaring migratory birds [58] and insects [59] which use the energy of air currents to propel them in turbulent air. Recent studies have demonstrated that fish may reduce locomotory costs by exploiting the energy of vortices generated by water moving past physical structures [36,57,60]. As such, the low ODBA values observed in the turning basins may have resulted from fish using the energy of vortices to hold position and propel their bodies, decreasing acceleration and consequently ODBA.

In this study there was considerable variability among individuals in terms of the total time taken to successfully ascend the fishway, which is linked to the physiological capacity or path selectivity of individual fish. Indeed the individual ability of fish to successfully negotiate a fishway is a result of the interaction between endogenous (i.e. physiology, motivation) and exogenous factors (i.e. hydraulics, temperature, pH) [61]. Hydraulics have been shown to strongly affect fish swimming performance, fish migration patterns [36,37] and successful fish passage. The behavioural response of individual fish to the hydrodynamic heterogeneity of flows strongly depends on the changing magnitude of spatial and temporal forces acting on the fish, the time of fish exposure, species, life stage and individual size [62,63]. Thus, fishways that are uniformly successful at passing all individuals or species are rare [64]. Quantifying the physiological consequences of passage of different fish species through specific types of fishways is therefore important in particular for species of poor swimming performance. Silver redhorse spent significantly longer times in the turning basins (TIB) when compared to the time spent in regular basins. Turning basins are designed to meet physical (hydraulic) and biological criteria to reduce flow velocity and provide resting areas for fish; yet it has been demonstrated that turning basins can also “trap” fish, drastically increasing the total passage times through fishways [65]. Considering that fishways should be designed to ensure fish passage with minimum delay, from the result obtained in the present study, the hydrodynamic environments within turning basins may be considered a feature that is a constraint rather than a benefit. This corroborates the findings from Thiem 2013 [66], whereby the swimming activity and energetic costs of adult lake sturgeon during fishway passage were studied and the author concluded that the absence of turning basins in vertical slot fishways could reduce sturgeon passage time. In the present study fish also spent longer times in the most upstream pool when compared with the time spent in the other regular basins. This behaviour could have occurred due to the changes of the hydrodynamics between this basin and the end of the fishway caused by the installation of the trap. The different tactile (water velocity, acceleration) cues encountered by fish might have been perceived as natural hazards and therefore may have induced an avoidance reaction [67]. The evidence of the learning ability of fish is widespread and dates back to the late 1800s [67]. The learning process, which refers to a change in behaviour with experience [68] was also evident in this study as TIB was observed to decrease with the number of attempts made by fish to ascend the fishway. In this case, the hydraulic cues associated with each basin may have played a decisive role on fish movement. Indeed it is known that many fish species can learn spatial patterns and use landmarks to navigate during migration [69]. Fish that learn the best route to ascend the fishway are more likely to succeed in passing the structure with minimum delays and energetic expenditure associated, decreasing the associated risk of passage through fishways.

Overall, fish exhibited low GS in the turning basins and in the last basin and high GS in the regular basins, in particular, in the basins immediately upstream of each turning basin. Moreover ODBA was relatively low until fish reached the first turning basin, after which the energetic activity of the silver redhorse was found to continuously increase. The former results may have occurred because fish experienced an abrupt increase in flow velocity transitioning from a turning basin to a regular basin [54] and may have switched to an anaerobic swimming mode of short duration. High speed swimming under anaerobic conditions has been shown to occur during passage through basins within fishways where velocities are highest [13]. Anaerobic swimming modes are mechanical and physiological/chemical processes that involve high energetic expenditures which may explain the increase in ODBA after the first turning basin. Anaerobic swimming cannot be sustained for long periods so it is unlikely that redhorse exhibited anaerobiosis during the entirety of the ascent. Presumably silver redhorse, which are not known for their swimming ability [39] were able to select paths that did not require anaerobiosis except when transitioning from the turning basin to regular fishway pools.

Exogenous factors such as length of the fishways [70] and fishway steepness [71] affect the physiological and behavioural response of fish by controlling the hydrodynamic conditions fish are exposed to during passage. Nevertheless, in this study the exogenous factors within the fishway had less influence on fish than the endogenous factors as there was no relationship between TIB and ODBA, and TIPB and ODBAPB. This result provides evidence that endogeneous conditions (motivation, morphology, energetic reserves) play a determinant role in fish capacity to successfully pass through fishways, despite the similarity of hydraulic conditions among basins of similar geometries, distinct behavioural and physiological responses were observed.

The use of accelerometry in this study provides an explicit illustration of the practical applicability of biologging tools to inform water resource development specifically in relation to fishway design. The use of biologging tools enables increased knowledge and understanding of the behaviour and physiological effort of fish during fishway passage. With this type of comprehensive information, biologists and engineers may be able to use accelerometry techniques to determine where and how infrastructure can be modified to maximize fish migration whilst minimizing energetic activity. For example, results from this study demonstrate that turning basins delay silver redhorse upstream passage. Thus future fishway designs focused in this species should consider avoiding this type of pool, whilst balancing the requirements of other species. In future improvements of existent fishways, the above situation may be minimized by using retrofit structures to break long eddies minimizing potential fish disorientation and improving free passage with minimum delay [36, 53].

Future studies that integrate biologging tools to analyse behaviour and physiological effort of fish during fishway passage are imperative to provide a better understanding of the energetic impact of fishways on the total energetic budget, migratory capacity and reproductive success of fish. Such studies should be coupled with similar migratory studies over natural barriers to migration so that comparisons can be made between populations that are required to ascend anthropogenic and natural obstacles to migration.

## Supporting Information

S1 Dataset (XLS)

S2 Dataset (XLS)

S1 File Explanation of data included in supplementary information S1 and S2. (DOC)

## References

[1] Hinch SG, Bratty J. Effects of Swim Speed and Activity Pattern on Success of Adult Sockeye Salmon Migration through an Area of Difficult Passage. Trans. Am. Fish. Soc. 2000; 129: 589–606. 10.1577/1548-8659(2000)129,lt0598:eossaa,gt2.0.co2

[2] Peake S, Beamish F, McKinley R. Relating swimming performance of lake sturgeon, *Acipenser fulvescens*, to fishway design. Can. J. Fish. Aquat. Sci. 1997; 54: 1361–1366. 10.1139/cjfas-54-6-1361

[3] Bunt CM. Fishways for warm water species: utilization patterns, attraction efficiency, passage efficiency and relative physical output. Ph.D. Dissertation, University of Waterloo. 1999.

[4] Cooke SJ, Hinch SG. Improving the reliability of fishway attraction and passage efficiency estimates to inform fishway engineering, science, and practice. Ecol Eng. 2013; 58: 123–132. 10.1016/j.ecoleng.2013.06.005

[5] Roscoe DW, Hinch SG, Cooke SJ, Patterson DA. Fishway passage and post-passage mortality of up-river migrating sockeye salmon in the Seton River, British Columbia. River Res Applic. 2011; 27: 693–705. 10.1002/rra.1384

[6] Fulton CJ, Johansen JL, Steffensen JF. Energetic Extremes in Aquatic Locomotion by Coral Reef Fishes. PLoS ONE. 2013; 8, e54033 10.1371/journal.pone.0054033 23326566 PMC3541231

[7] Bosclair D, Leggett WC. The Importance of Activity in Bioenergetics Models Applied to Actively Foraging Fishes. Can J Fish Aquat Sci. 1989; 46: 1859–1867. 10.1139/f89-234

[8] Ohlberger J, Staaks G, van Dijk PLM, Hölker F. Modelling energetic costs of fish swimming. J Exp Zool A Comp Exp Bio. 2005; 303: 657–664. 10.1002/jez.a.181 16013050

[9] Lucas M, Baras E, Thom T, Duncan A, Slavík O. Migration of Freshwater Fishes, Blackwell Science Ltd, Oxford, UK 2001.

[10] McElroy B, DeLonay A, Jacobson R. Optimum swimming pathways of fish spawning migrations in rivers. Ecology. 2012; 93, 29–34. 22486084 10.1890/11-1082.1

[11] Standen EM, Hinch SG, Healey MC, Farrell AP. Energetic costs of migration through the Fraser River Canyon, British Columbia, in adult pink (*Oncorhynchus gorbuscha*) and sockeye (*Oncorhynchus nerka*) salmon as assessed by EMG telemetry. Can. J. Fish. Aquat. Sci. 2002; 59: 1809–1818. 10.1139/f02-151

[12] Cooke SJ, Hinch SG, Wikelski M, Andrews RD, Kuchel LJ, Wolcott TG, et al Biotelemetry: a mechanistic approach to ecology. Trends Ecol Evol. 2004; 19: 334–343. 10.1016/j.tree.2004.04.003 16701280

[13] Alexandre CM, Quintella BR, Silva AT, Mateus CS, Romão F, Branco P, et al Use of electromyogram telemetry to assess the behavior of the Iberian barbel (*Luciobarbus bocagei* Steindachner, 1864) in a pool-type fishway. Ecol Eng. 2013; 51: 191–202. 10.1016/j.ecoleng.2012.12.047

[14] Wilson RP, Quintana F, Hobson JV Construction of energy landscapes can clarify the movement and distribution of foraging animals. Proc. R. Soc. B. 2012; 279: 975–980. 10.1098/rspb.2011.1544 21900327 PMC3259934

[15] Morales JM, Moorcroft PR, Matthiopoulos J, Frair JL, Kie JG, Powell RA, et al Building the bridge between animal movement and population dynamics. Philos Trans R Soc Lond B Biol Sci. 2010; 365: 2289–2301. 10.1098/rstb.2010.0082 20566505 PMC2894961

[16] McNab B. The Physiological Ecology of Vertebrates: A View from Energetics, Publisher: Cornell University Press 2002.

[17] Tomlinson S, Arnall SG, Munn A, Bradshaw SD, Maloney SK, Dixon KW, et al Applications and implications of ecological energetics. Trends Ecol. Evol. 2014; 29: 280–290. 10.1016/j.tree.2014.03.003 24725438

[18] Payne NL, Taylor MD, Watanabe YY, Semmens JM. From physiology to physics: are we recognizing the flexibility of biologging tools? J Exp Biol. 2014; 217: 317–322. 10.1242/jeb.093922 24477606

[19] Gleiss AC, Gruber SH, Wilson RP. Multi-channel data-logging: towards determination of behaviour and metabolic rate in free-swimming sharks In Tagging and Tracking of Marine Animals with Electronic Devices Nielsen JL, Arrizabalaga H, Fragoso N, Hobday A, Lutcavage M, Sibert J (Eds.) Springer, New York 2009.

[20] Yoda K, Naito Y, Sato K, Takahashi A, Nishikawa J, Ropert-Coudert Y, et al A new technique for monitoring the behaviour of free-ranging Adélie penguins. J Exp Biol. 2001; 204: 685–90. 11171350 10.1242/jeb.204.4.685

[21] Halsey LG, Green JA, Wilson RP, Frappell PB. Accelerometry to Estimate Energy Expenditure during Activity: Best Practice with Data Loggers Physiol Biochem Zool. 2009; 82: 396–404. doi: 101086/589815 19018696 10.1086/589815

[22] Bouten C, Koekkoek KT, Verduin M, Kodde R, Janssen JD. A triaxial accelerometer and portable data processing unit for the assessment of daily physical activity. IEEE Trans Biomed Eng. 1997; 4:136–147. 10.1109/10.554760 9216127

[23] Halsey LG, Shepard ELC, Wilson RP. Assessing the development and application of the accelerometry technique for estimating energy expenditure Comp Biochem Physiol A Mol Integr Physiol. 2011; 158: 305–314. doi: 101016/jcbpa201009002 20837157 10.1016/j.cbpa.2010.09.002

[24] Sato K, Naito Y, Kato A, Niizuma Y, Watanuki Y, Charrassin JB, et al Buoyancy and maximal diving depth in penguins: do they control inhaling air volume? J Exp Biol. 2002; 205: 1189–97 11948196 10.1242/jeb.205.9.1189

[25] Robson AA, Chauvaud L, Wilson RP, Halsey LG. Small actions, big costs: the behavioural energetics of a commercially important invertebrate J R Soc Interface. 2012; 9: 1486–1498. doi: 101098/rsif20110713 22219397 10.1098/rsif.2011.0713 PMC3367807

[26] Shepard E, Wilson R, Halsey L, Quintana F, Laich AG, Gleiss A, et al Derivation of body motion via appropriate smoothing of acceleration data Aquatic Biol. 2008; 4: 235–241. doi: 103354/ab00104

[27] Lyons GN, Halsey LG, Pope EC, Eddington JD, Houghton JDR. Energy expenditure during activity in the American lobster Homarus americanus: Correlations with body acceleration Comp Biochem Physiol A Mol Integr Physiol. 2013; 166: 278–284. doi: 101016/jcbpa201306024 23811045 10.1016/j.cbpa.2013.06.024

[28] Shepard E, Wilson R, Quintana F, Laich AG, Liebsch N, Da Albareda, et al Identification of animal movement patterns using tri-axial accelerometry. Endanger Species Res. 2010; 10: 47–60. doi: 103354/esr00084

[29] Gleiss AC, Jorgensen SJ, Liebsch N, Sala JE, Norman B, Hays GC, et al Convergent evolution in locomotory patterns of flying and swimming animals. Nat Commun. 2011; 2: 352 doi: 101038/ncomms1350 21673673 10.1038/ncomms1350

[30] Green JA, Halsey LG, Wilson RP, Frappell PB. Estimating energy expenditure of animals using the accelerometry technique: activity, inactivity and comparison with the heart-rate technique. J Exp Biol. 2009; 212: 745–746. doi: 101242/jeb030049 10.1242/jeb.026377 19181894

[31] Shepard ELC, Wilson RP, Quintana F, Laich AG, Forman DW. Pushed for time or saving on fuel: fine-scale energy budgets shed light on currencies in a diving bird. Proc R Soc B. 2009; 276: 3149–3155. doi: 101098/rspb20090683 19515661 10.1098/rspb.2009.0683 PMC2817130

[32] Gleiss AC, Wilson RP, Shepard ELC. Making overall dynamic body acceleration work: on the theory of acceleration as a proxy for energy expenditure. Methods Ecol Evol. 2011; 2: 23–33. doi: 101111/j2041-210X201000057x

[33] Gleiss AC, Dale JJ, Holl KN, Wilson RP, Holland KN. Accelerating estimates of activity-specific metabolic rate in fishes: Testing the applicability of acceleration data-loggers. J Exp Mar Biol Ecol. 2010; 385: 85–91. doi: 101016/jjembe201001012

[34] Hasler CTHT, Pon LBPB, Roscoe DWRW, Mossop B, Patterson DAPA, Hinch SGHG, et al Expanding the ‘toolbox’ for studying the biological responses of individual fish to hydropower infrastructure and operating strategies. Env Rev. 2009; 17: 179–197. doi: 101139/a09-008

[35] Young JL, Bornik ZB, Marcotte ML, Charlie KN, Wagner GN, Hinch SG, et al Integrating physiology and life history to improve fisheries management and conservation. Fish Fish. 2006; 7: 262–283. doi: 101111/j1467-2979200600225x

[36] Silva AT, Katopodis C, Santos JM, Ferreira MT, Pinheiro AN. Cyprinid swimming behaviour in response to turbulent flow. Ecol Eng. 2012; 44: 314–328. doi: 101016/jecoleng201204015

[37] Silva AT, Santos JM, Ferreira MT, Pinheiro AN, Katopodis C. Effects of water velocity and turbulence on the behaviour of Iberian barbel (*Luciobarbus bocagei*, Steindachner 1864) in an experimental pool-type fishway. River Res Applic. 2011; 27: 360–373. doi: 101002/rra1363

[38] Butler P, Green J, Boyd IL, Speakman JR. Measuring metabolic rate in the field: the pros and cons of the doubly labelled water and heart rate methods. Funct Ecol. 2004; 18: 168–183. 10.1111/j.0269-8463.2004.00821.x

[39] Hatry C, Thiem JD, Binder TR, Hatin D, Dumont P, Stamplecoskie KM, et al Comparative Physiology and Relative Swimming Performance of Three Redhorse (*Moxostoma* spp) Species: Associations with Fishway Passage Success. Physiol Biochem Zool. 2014; 87: 148–159. doi: 101086/671900 24457929 10.1086/671900

[40] Cooke SJ, Bunt CM, Hamilton SJ, Jennings CA, Pearson MP, Cooperman MS, et al Threats, conservation strategies, and prognosis for suckers (Catostomidae) in North America: insights from regional case studies of a diverse family of non-game fishes. Biol Conserv. 2005; 121:317–331. 10.1016/j.biocon.2004.05.015

[41] Walsh SJ, Haney DC, Timmerman CM, Dorazio RM. Physiological tolerances of juvenile robust redhorse, *Moxostoma robustum*: conservation implications for an imperiled species. Env Biol Fish. 1998; 51: 429–444. 10.1023/A:1007486632102

[42] Thiem J, Binder T, Dawson J, Dumont P, Hatin D, Katopodis C, et al Behaviour and passage success of upriver-migrating lake sturgeon *Acipenser fulvescens* in a vertical slot fishway on the Richelieu River, Quebec, Canada. Endanger Species Res. 2011; 15: 1–11. doi: 103354/esr00360

[43] Collins MR, Cooke DW, Smith TIJ, Post WC, Russ DC, Walling DC. Evaluation of four methods of transmitter attachment on shortnose sturgeon, *Acipenser brevirostrum* . J Appl Ichthyol. 2002; 18: 491–494. 10.1046/j.1439-0426.2002.00386.x

[44] Winter JD. Underwater biotelemetry In: Nielsen L. A. and Johnson D. L., editors. Fisheries techniques. American Fisheries Society, Bethesda, Maryland 1983 pp. 371–395.

[45] Brown RS, Cooke SJ, Anderson WG, McKinley RS. Evidence to challenge the “2% Rule” for biotelemetry. N. Am. J. Fish. Manage.1999; 19: 867–871 10.1577/1548-8675(1999)019<0867:ETCTRF>2.0.CO;2

[46] Bunt CM, Cooke SJ. Post-spawn movements and habitat use of greater redhorse, Moxostoma valenciennesi. Ecol Freshw Fish. 2001; 10:57–60. doi: 10.1111/j.1600 0633.2001.tb00194.x

[47] Zuur A, Ieno E, Meesters E. A Beginner's Guide to R. 2009.

[48] Pinheiro J, Bates D. Nonlinear mixed-effects models. 2012. Available: ftp://ftp.uni-bayreuth.de/pub/math/statlib/R/CRAN/doc/packages/nlme.pdf

[49] Burnham KP, Anderson DR. Model Selection and Multimodel Inference Springer Science, Business Media 2002.

[50] White H. A heteroskedasticity-consistent covariance matrix estimator and a direct test for heteroskedasticity. Econometrica. 1980; 48: 817–838.

[51] Cleasby IR, Nakagawa S. Neglected biological patterns in the residuals. Behav Ecol Sociobiol. 2011; 65: 2361–2372. doi: 101007/s00265-011-1254-7

[52] Wright S, Metcalfe JD, Hetherington S, Wilson R. Estimating activity-specific energy expenditure in a teleost fish, using accelerometer loggers. Mar Ecol Prog Ser. 2014; 496:19–32. 10.3354/meps10528

[53] Burnett NJ, Hinch SG, Braun DC, Casselman MT, Middleton CT, Wilson SM, et al Burst swimming in areas of high flow: delayed consequences of anaerobiosis in wild adult sockeye salmon. Physiol. Biochem. Zool. 2014; 87:587–98. 10.1086/677219 25244372

[54] Marriner BA, Baki ABM, Zhu DZ, Thiem JD, Cooke SJ, Katopodis C. Field and numerical assessment of turning pool hydraulics in a vertical slot fishway. Ecol Eng. 2014; 63: 88–101. doi: 101016/jecoleng201312010

[55] Pope SB. Turbulent Flows Cambridge University Press 2000.

[56] Lacey RWJ, Neary VS, Liao JC, Enders EC, Tritico HM. The IPOS framework: linking fish swimming performance in altered flows from laboratory experiments to rivers. River Res Applic. 2012; 28: 429–443. doi: 101002/rra15849

[57] Liao JC.A review of fish swimming mechanics and behaviour in altered flows. Philos Trans R Soc Lond B Biol Sci. 2007; 362: 1973–1993. doi: 101098/rstb20072082 17472925 10.1098/rstb.2007.2082 PMC2442850

[58] Hedenstrӧm A. Migration by soaring or flapping flight in birds: the relative importance of energy cost and speed. Philos Trans R Soc Lond B Biol Sci. 1993; 342: 353–361. doi: 101098/rstb19930164

[59] Ristroph L, Bergou AT, Guckenheimer J, Wang ZJ, Cohen I. Paddling Mode of Forward Flight in Insects. Phys Rev Lett. 2011; 106: 178103 doi: 101103/PhysRevLett106178103 21635066 10.1103/PhysRevLett.106.178103

[60] Przybilla A, Kunze S, Rudert A, Bleckmann H, Brucker C. Entraining in trout: a behavioural and hydrodynamic analysis. J Exp Biol.2010; 213: 2976–2986. doi: 101242/jeb041632 20709926 10.1242/jeb.041632

[61] Castro-Santos T, Cotel A, Webb PW. Fishway evaluations for better bioengineering: an integrative approach In Challenges for Diadromous Fishes in a Dynamic Global Environment, Haro A, Smith RA, Rulifson CM, Moffit RJ, Klauda MJ, Dadswell RA, Cunjak JE, Cooper KL, Beal TS Avery (eds) American Fisheries Society Symposium: Bethesda, MD 2009.

[62] Cotel A, Webb P, Tritico H. Do Brown Trout Choose Locations with Reduced Turbulence? T am fish soc.2006; 135: 610–619. doi: 101577/T04-1961

[63] Lupandin AI. Effect of Flow Turbulence on Swimming Speed of Fish. Biol Bull. 2005; 32: 461–466. 16240752

[64] Bunt CM, Castro-Santos T, Haro A. Performance of fish passage structures at upstream barriers to migration. River Res Applic 2012; 28: 457–478. doi: 101002/rra1565

[65] Tarrade L, Texier A, David L, Larinier M. Topologies and measurements of turbulent flow in vertical slot fishways. Hydrobiologia. 2008; 609: 177–188. doi: 101007/s10750-008-9416-y

[66] Thiem J. Behaviour and energetics of sturgeon fishway passage. Ph.D. Dissertation, Carleton University, Ottawa, Ontario, Canada. 2013.

[67] Kieffer J, Colgan P.The role of learning in fish behaviour. Rev fish biol fisher. 1992; 2: 125–143. doi: 101007/BF00042881

[68] Dill LM. Adaptive Flexibility in the Foraging Behavior of Fishes. Can J Fish Aquat Sci. 1983; 40: 398–408. doi: 101139/f83-058

[69] Odling-Smee L, Braithwaite VA. The role of learning in fish orientation. Fish Fish. 2003; 4: 235–246. doi: 101046/j1467-2979200300127 14611652

[70] Noonan MJ, Grant JW, Jackson CD. A quantitative assessment of fish passage efficiency. Fish and Fisheries. 2012; 13:450–464. 10.1111/j.1467-2979.2011.00445.x

[71] Mallen-Cooper M, Brand DA. Non-salmonids in a salmonid fishway: what do 50 years of data tell us about past and future fish passage? Fish Manag Ecol. 2007; 14: 319–332. 10.1111/j.1365-2400.2007.00557.x

