# Supplementary material for: Behaviour and Locomotor Activity of a Migratory Catostomid during Fishway Passage
Source: PLoS One. 2015 Apr 8;10(4):e0123051. doi: 10.1371/journal.pone.0123051 (PMC4390351; doi:10.1371/journal.pone.0123051)
Supplement: S1 File — (DOC) [file pone.0123051.s003.doc]

**S3 Supporting information.**

**Explanation of data included in supplementary information S1 and S2**

LIST OF VARIABLES:

ID – fish identity number

ID2 – fish tag number

TL- total length of the fish (mm)

W – weight of the fish (kg)

ATMP- attempt number

BAS- basin

TBAS- time in basin

GS- groundspeed

TIPB – time in previous basin

GSPB – groundspeed in previous basin

ODBA – overall dynamic body acceleration

ODBAPB- overall body dynamic acceleration in previous basin
